# Degradation of switchgrass by *Bacillus subtilis* 1AJ3 and expression of a beta-glycoside hydrolase

**DOI:** 10.3389/fmicb.2022.922371

**Published:** 2022-07-29

**Authors:** Lingling Ma, Xin Wang, Jingwen Zhou, Xin Lü

**Affiliations:** ^1^Science Center for Future Foods, Jiangnan University, Wuxi, China; ^2^Key Laboratory of Industrial Biotechnology, Ministry of Education and School of Biotechnology, Jiangnan University, Wuxi, China; ^3^Laboratory of Bioresources, College of Food Science and Engineering, Northwest A&F University, Xianyang, China

**Keywords:** *B. subtilis*, *s*witchgrass, lignocellulose degradation substrate, therm-tolerant bacteria, acid-heat treatment, beta-glycoside hydrolase, molecular docking, coffee grounds

## Abstract

Increasing demand for carbon neutrality has led to the development of new techniques and modes of low carbon production. The utilization of microbiology to convert low-cost renewable resources into more valuable chemicals is particularly important. Here, we investigated the ability of a cellulolytic bacterium, *Bacillus subtilis* 1AJ3, in switchgrass lignocellulose degradation. After 5 days of culture with the strain under 37°C, cellulose, xylan, and acid-insoluble lignin degradation rates were 16.13, 14.24, and 13.91%, respectively. Gas chromatography–mass spectrometry (GC-MS) analysis and field emission scanning electron microscopy (FE-SEM) indicated that the lignin and surface of switchgrass were degraded after incubation with the bacterial strain. Strain 1AJ3 can grow well below 60°C, which satisfies the optimum temperature (50°C) condition of most cellulases; subsequent results emphasize that acid-heat incubation conditions increase the reducing sugar content in a wide range of cellulosic biomass degraded by *B. subtilis* 1AJ3. To obtain more reducing sugars, we focused on β-glycoside hydrolase, which plays an important role in last steps of cellulose degradation to oligosaccharides. A β-glycoside hydrolase (Bgl-16A) was characterized by cloning and expression in *Escherichia coli* BL21 and further determined to belong to glycoside hydrolase (GH) 16 family. The Bgl-16A had an enzymatic activity of 365.29 ± 10.43 U/mg, and the enzyme’s mode of action was explained by molecular docking. Moreover, the critical influence on temperature (50°C) of Bgl-16A also explained the high-efficiency degradation of biomass by strain under acid-heat conditions. In terms of potential applications, both the strain and the recombinant enzyme showed that coffee grounds would be a suitable and valuable substrate. This study provides a new understanding of cellulose degradation by *B. subtilis* 1AJ3 that both the enzyme action mode and optimum temperature limitation by cellulases could impact the degradation. It also gave new sight to unique advantage utilization in the industrial production of green manufacturing.

## Introduction

The energy transition is a key turning toward carbon neutrality, which benefits developing countries’ environment, climate, and economy (De La Peña et al., 2022). To achieve energy transition and low carbon production, the most important part is to move from fossil fuels to renewable energy sources. Plant-based lignocellulosic materials have become the main substrate for biofuels and bioconversion products, as their multi-carbon component and their derivative can be transformed into solid or liquid base material for further syntheses, such as sugars, alcohols, lipids, and furfural (Bendeddouche et al., 2020). The biofuel industry is an important area for further utilization of biomass lignocellulose. Biomass, including multiple plants, crops, microalgae (Nagappan et al., 2021), and process by-products or wastes, has the most abundant area on earth and has renewable properties that make it a major feedstock for biofuels (Kaloudas et al., 2021). Switchgrass, suited for biofuel production due to its high energy reserves and low planting cost (Sanderson et al., 1996; Parrish and Fike, 2005; Bouton, 2007), has been widely used to study biomass degradation (Poovaiah et al., 2015), assess liquid fuels (McLaughlin et al., 2006; Wu et al., 2006), and study molecular breeding (Bouton, 2007). Moreover, other readily abandoned available cellulosic materials, like wheat straw and corn straw, could be cellulosic substrates for degradation.

Saccharification, the conversion of lignocellulose into reduced sugar, is always the key process limiting factor of the rate and yield of biofuel conversion. The lignocellulose structure makes it hard to hydrolyze, particularly when hemicellulose and lignin are present, as they prevent cellulose–cellulase direct interaction (Houfani et al., 2020). Pretreatment might weaken the tough structure of lignocellulose, making cellulose more accessible to reducing sugar (Karimi and Taherzadeh, 2016). A combination of pretreatment methods, including physical, chemical, and biological, is commonly utilized in industrial processes (Chaturvedi and Verma, 2013). Biological methods have gained more interest in the past few years for their potential degradation ability. Bacteria attracted more attention for their abundant enzyme system, fast growth, pressure resistance, and ease of genetic manipulation. Moreover, different types of cellulose may induce bacteria to produce different cellulases, making microbes lignocellulose-source-specific. Thus, a widely biomass substrate-degraded bacteria would be preferred.

**FIGURE 1:**
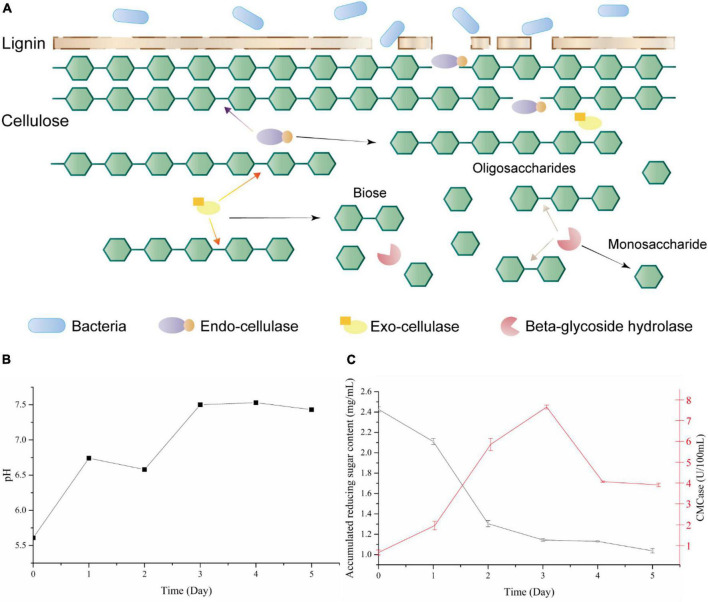
**(A)** Lignocellulose degradation mode by bacteria and cellulases; **(B)** pH changes among bacteria 1AJ3 cultural process; **(C)** accumulation of reducing sugar (black) and CMCase activity (red) of switchgrass cultured with strain 1AJ3 for 5 days.

Cellulases consist of an enzyme system for cellulose degradation, divided into three classes: endo-cellulases, exo-cellulases, and beta-glycoside hydrolase based on their action mode (Figure 1A). Both endo- and exo-cellulases can hydrolyze long cellulose chains into shorter chains or oligosaccharides; the difference is that endo-cellulase hydrolyzes at random, while exo-cellulase can only hydrolyze cellobiose from the end of the cellulose chain. After that, beta-glycoside hydrolases hydrolyze oligosaccharides into monosaccharide which provide materials for further value-added products (Han and Chen, 2008). So, beta-glycoside hydrolases play an important role in the complete hydrolysis of cellulose to monosaccharide, and also being a rate-limiting step. Moreover, beta-glycoside hydrolase affects various biodegradation processes, such as the food (An et al., 2017), feedstuff, and biofuel (Liew et al., 2018) industries. Bacteria are an abundant source of glycoside hydrolase, especially *Bacillus* species, which displayed huge application potential in industry (Chamoli et al., 2016).

In our previous study, a cellulolytic bacterium, *B. subtilis* 1AJ3, was isolated from rotten woods of the Qinling Mountains. The strain could grow using sodium carboxymethyl cellulose (CMC-Na) and Avicel as carbon sources and theoretically can degrade cellulose in biomass. This study evaluated the specific degradation capacity in biomass by 1AJ3 strain. Then, since the temperature at which the bacteria grew did not correspond to the optimal temperature for cellulase activity, an increase in the incubation temperature factor was considered to improve the saccharification rate. To better understand the limiting factor during the cellulose degradation process, we focused on the last steps’ hydrolyzation enzymes, beta-glycoside hydrolases, that hydrolyze oligosaccharides into monosaccharide. A recombinant beta-glycoside hydrolase from strain *B. subtilis* 1AJ3 was characterized by cloning and expression in *Escherichia coli* BL21 (DE3). Also, the theoretical mechanism of Bgl-16A was studied by substrate molecular docking. Our results showed that beta-glycoside hydrolase would be a limiting factor for cellulose degradation by *B. subtilis* strain 1AJ3 due to its hydrolytic activity mode of action and strict action temperature. Furthermore, we also found that coffee grounds can be a potential substrate for biofuels in which bacterial strains or beta-glycoside hydrolase can degrade.

## Materials and methods

### Strain, plasmid, and medium

*Bacillus subtilis* 1AJ3 (GenBank No. MG062801) was isolated from rotten wood in Qinling mountains (Ma et al., 2020c). The bacteria were grown in LB medium (NaCl 10 g/L, tryptone 10 g/L, yeast extract powder 5 g/L), single carbon source medium of switchgrass (7%), and ionic liquids of (NH_4_)_2_SO_4_ 2.0 g/L, MgSO_4_7H_2_O 0.5 g/L, and K_2_HPO_4_ 1.0 g/L at pH of 7.2 or 5.6. The medium was sterilized at 121°C for 20 min before use.

Plasmid pET-28a was used to construct a recombinant expression vector. *E. coli* BL21(DE3) was used as the expression host. Restriction endonucleases *Nco*I and *Xho*I were purchased from Takara. DNA Extraction Kit, Plasmid Extraction Kit, and PCR Purification Kit were purchased from Omega Bio-Tek, Inc. Primer synthesis and sequencing were entrusted to Sangon Biotech Inc. (Shanghai).

### Determination of reducing sugar content and cellulases activity

Reducing sugar content in the culture medium was determined by the DNS method (Miller, 1959). CMCase activity was analyzed as previously described (Yang et al., 2014). One unit (U) of CMCase refers to the amount of enzyme that produces 1 μmol glucose in 1 min under a specific pH and temperature.

The recombinant beta-glycoside hydrolase activity was determined using p-NPG (4-Nitrophenyl β-D-galactopyranoside, CAS: 3150-24-1) as substrate at 50°C for 10 min with p-NP (*p*-Nitrophenol, CAS: 100-02-7) as standard (Goswami et al., 2016). One unit (U) of the beta-glycoside hydrolase was defined as the amount of enzyme required to release 1 μmol of p-NP per minute.

### Hydrolyzation and analysis of switchgrass by strain *Bacillus subtilis* 1AJ3

*Bacillus subtilis* 1AJ3 was grown in CMC-Na liquid medium at 37°C, 150 rpm for 48 h. The bacteria were collected by centrifugation at 10,000 × *g* for 10 min at 4°C and resuspended in sterile culture medium ionic liquid at OD_600nm_ 1.0. Inoculation was done by transferring 10% of cells into the switchgrass medium and incubating at 37°C, 150 rpm, for 5 days. pH was monitored daily. Blank was set as the inoculation medium without bacteria.

The culture was divided into two for analysis: the culture liquid and the insoluble residuum. Culture liquid was separated from the culture media by centrifugation at 12,000 × *g*. The switchgrass residuum was filtered from the sand core fuel, rinsed with distilled water, and oven-dried at 60°C overnight. The amount of total reducing sugar content in the culture liquid was determined by DNS, as described before (Miller, 1959). Cellulosic composition analysis of the culture liquid and switchgrass residuum was done using acid hydrolysis (Sluiter et al., 2008, 2012) following guidelines of National Renewable Energy Laboratory (NREL) guidelines to detect degraded low molecular sugars and calculate the cellulose and xylan degradation levels in the culture liquid and dry switchgrass residuum. The results are shown as the “mean ± standard deviation” of three independent experiments.

### Field-emission scanning electron microscope and gas chromatography–mass spectrometry assay

**FIGURE 2:**
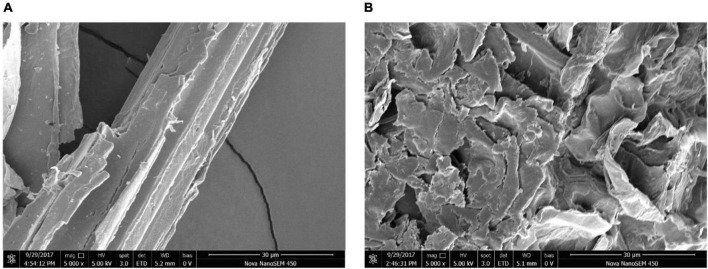
Field emission scanning electron microscopy images before and after degradation of strains of switchgrass. **(A)** For blank sample; **(B)** for degraded sample.

The degraded switchgrass sample cultured with *B. subtilis* 1AJ3 (Figure 2) for 5 days and the blank sample were analyzed by field-emission scanning electron microscope (FE-SEM) to visualize lignocellulose hydrolyzation by the bacteria (Kuuskeri et al., 2016). Gas chromatography–mass (GC-MS) spectrometry was used to evaluate switchgrass lignocellulose hydrolyzation by the bacteria. FE-SEM and GC-MS were performed as described before (Yang et al., 2018). The control was treatment without bacteria. The presence of benzene ring compounds was verified against the NIST library based on retention times (RTs).

### *Bacillus subtilis* 1AJ3 thermostability and its applications in various cellulosic materials

High temperature is needed during industrial production and enzyme reaction processing, and the optimal temperature for cellulase activity is 50°C. *B. subtilis* 1AJ3 was allowed to grow under different temperatures, and the highest tolerable temperature was recorded. Briefly, *B. subtilis* 1AJ3 was incubated at 60, 80, and 100°C for 2 days by coating the suspension on plates. Bacterial colonies number and growth statements on the plates (solid medium containing Congo-red) would be observed after 24 and 48 h later.

To identify a suitable biomass cellulose substrate *B. subtilis* 1AJ3, the bacteria’s ability to degrade 10 types of biomass cellulose materials, switchgrass, wheat straw, corn stalks, corn cob, rice husk, sugarcane stalk, pea straw, ginger stems and leaves, peanut shells, and coffee grounds, was examined. Raw biomass cellulose was crushed, dried at 60°C, and weighed. The weighed samples were incubated at 100°C for 2 h and added to the bacteria suspension to minimize contamination. The bacteria were incubated in an LB medium, and the cells were collected by centrifugation at 10,000 × *g* for 10 min at 4°C. Cells were washed using ion liquid at different pHs and resuspended to an OD_600nm_ = 1.0 for further analysis.

Previously, it has been shown that an initial medium pH of 4.0 provides a higher reducing sugar content (Ma et al., 2020a). *B. subtilis* 1AJ3 could resist of pH 4.0 and 80^°^C growing temperature, and consider of most cellulases’ optimal temperature is 50°C, the bacteria’s biomass cellulose degradation was evaluated in two conditions: Condition 1: initial medium pH 7.0, at 37°C, 150 rpm for 72 h. Condition 2: initial medium pH 4.0, at 50°C, 150 rpm for 72 h. The degradation state and reducing sugar content were evaluated. The treatment without no strain was set as blank.

### Expression and purification of the recombinant Bgl-16A in *Escherichia coli*

A beta-glycoside hydrolase gene, *bgl*-16A, from *B. subtilis* 1AJ3 was amplified from the genomic DNA via polymerase chain reaction (PCR), using primers according to the primary study (Ma et al., 2020c). The recombinant expression vector was constructed through double digestion (*Nco*I and *Xho*I) and ligation with T4 ligase of pET-28a(+) plasmid and PCR products. The recombinant Bgl-16A contained a His-tag on the C-terminal. The recombinant vector with sequenced *bgl*-16A was transferred into *E. coli* BL21 (DE3) competent cells by thermal shock at 42°C for 90 s.

The recombinant *E. coli* was cultured in LB liquid medium containing 100 μg/mL kanamycin on a rotary shaker (220 rpm) at 37°C. The recombinant Bgl-16A protein was induced with 0.4 mM of isopropyl-β-thiogalactopyranoside (IPTG) at an OD_600nm_ of 0.6 for 10 h at 25°C. Following induction, the supernatant was collected by centrifugation at 10,000 rpm for 10 min at 4°C and washed with 1 × PBS buffer solution (pH 7.2) twice. Cells were resuspended in 1 × PBS buffer solution, followed by ultrasonication for 20 min using a SCIENTZ-IID ultrasonic homogenizer (Ningbo Scientz Biotechnology Polytron Technologies Inc., Zhejiang, China). The cell debris was removed by centrifugation at 12,000 *g* for 20 min at 4°C. The supernatant was collected and applied to a Ni-NTA affinity chromatography column (GE Healthcare Bio-Sciences AB, Uppsala, Sweden). Unbound protein was washed away with 40 mM imidazole, and the recombinant his-tagged Bgl-16A protein was eluted with 200 mM imidazole in 1 × PBS buffer solution (pH 7.2). The eluted and the purified proteins were detected with 10% SDS-PAGE. Protein content was measured by BCA method.

### Biochemical characterization of Bgl-16A

The effect of pH on cellulase activity was determined by assaying for enzyme activity over the pH range of 2.4–11.0 at 50°C for 10 min using phosphate (pH 2.4–8.0) and Tris-HCl (pH 8.5–11.0) buffers. For the pH stability assay, the enzyme was incubated at 50°C in the different buffers for 30 min without substrate and subsequently measured for enzyme activity at 50°C for 10 min.

The effect of the reaction temperature was measured between 0 and 100°C (10°C intervals) at the maximum pH for 30 min. The thermal stability of the enzyme was assessed at the same temperatures as above for 30 min, and the enzyme activity was then measured under controlled conditions.

The effect of metal ions (Na^+^, K^+^, Cu^2+^, Mg^2+^, Zn^2+^, Mn^2+^, Fe^2+^, Fe^3+^, Ca^2+^, and [NH_4_]^+^) and chemicals sodium dodecyl sulfate (SDS) and ethylenediaminetetraacetic acid (EDTA), at concentrations of 10, 5, and 1 mM, on enzyme activity after pre-incubation for 10 min at room temperature was measured under standard conditions. The enzyme activity determined for the control was set as 100%, and the relative activity under each condition was recorded.

We determined the synergy effect between Bgl-16A and Cel-A, and a endo-cellulase has been cloned from strain 1AJ3 (Ma et al., 2020a). 1% CMC-Na, 1% Avicel, and filter paper were used as the substrates to detect the cellulase activities with a single or combined enzymes (V: V = 1: 1). The synergistic coefficient of an enzymatic reaction was defined as DS = U_*All*/_(U_1_ + U_2_ + …), when DS value > 1 means that the enzymes have a synergistic effect.

### Molecular docking with the substrate

The amino acid sequence of Bgl-16A was compared with the PDB database^1^ and NCBI and showed 99.08% identity with protein ID 3O5S, covering 86% of the sequence. The enzyme active site selection was according to protein 3O5S. The Bgl-16A structure was obtained via AlphaFold 2, and molecular docking was performed via AutoDock software.

The ligand molecules of p-NPG and cellobiose were drawn in ChemDraw software and initially optimized by Chem3D (MM2 module) with an RMS of 0.001. The protein was set as the molecular docking receptor. AutoDock Tools with AutoGrid 4 module were used to perform docking and defined E134 and E138 as active amino acid residues based on a previous study (Furtado et al., 2011). The AutoDock software completed 100 docking results and determined the most suitable ones.

### Switchgrass and coffee ground saccharification by beta-glycoside hydrolase

Lignocellulose switchgrass was crashed into a 40-mesh size, and coffee grounds were washed using distilled water. Lignocellulose switchgrass and coffee grounds were dried overnight at 60°C. The crude recombinant enzyme in buffer (pH 8.6) was saccharified with 5% (w/v) switchgrass at 50°C for 20 h. Three grades of crude enzyme density were set as 10, 20, and 40% (v/v). The saccharification percentage was subsequently calculated as follows (Dadheech et al., 2018): Saccharification (%) = Sugar contents (mg)/Substrate (mg) × 100.

### Data and statistical analysis

Each experiment was performed in triplicate, and the results are shown as average ± SD, calculated via Excel 2019 (Microsoft, Redmond, WA, United States). Origin 2018 (OriginLab, Inc., Northampton, MA, United States) was used to draw the figures.

## Results

### Changes of culture during switchgrass degradation process

*Bacillus subtilis* 1AJ3 was cultured with switchgrass for 5 days at 37°C, 150 rpm. During the switchgrass degradation, the culture’s pH stabilized at 7.5 from day 3 (Figure 1B). This analysis showed that strain can grow under acidic pH because the culture medium was initially acidic (Ma et al., 2020a). Moreover, the strain modified the culture environment’s pH, which benefits its growth.

CMCase activity in culture during the degradation process rose at the beginning and reached maximum activity (7.49 U/100 mL) on day 3 (Figure 1C); after 4 days, it declined and stabilized (3.94 U/100 mL). During co-culture, the bacteria degraded switchgrass to produce reducing sugar and consumed the reducing sugar for energy. Thus, the reducing sugar content is the net product of the two events. Total reducing sugar content (Figure 1C) initially exhibited a downward trend and stabilized on day 3. In the first 3 days, the bacteria consumed the reducing sugar to meet its own growth needs. After that, switchgrass degradation and stabilization of the bacteria’s metabolism began to stabilize reducing sugar levels to about 1.13 mg/mL.

### Switchgrass lignocellulose degradation by *Bacillus subtilis* 1AJ3

Switchgrass was cultured with *B. subtilis* 1AJ3 for 5 days, and the cellulose, hemicellulose (primarily xylan), and acid-insoluble lignin contents were monitored daily (Table 1). In addition, glucan and xylose contents in culture liquid were measured by HPLC, and the cellulose and xylan contents were calculated.

**TABLE 1:** Chemical degradation components of switchgrass by *Bacillus subtilis* 1AJ3.

Cultivation time (days)	Weight loss (%)	Cultural liquid composition^2^ (% wt/wt)		Dry matter composition (% wt/wt)		
		Cellulose	Xylan	Cellulose	Xylan	Acid-insoluble lignin
Blank^1^	21.22 ± 0.26	3.14 ± 0.09	1.09 ± 0.05	30.35 ± 0.48	16.14 ± 0.36	18.52 ± 0.31
1	23.61 ± 0.72	0.90 ± 0.01	1.20 ± 0.02	27.05 ± 0.04	15.61 ± 0.27	17.87 ± 0.10
2	23.79 ± 0.41	0.83 ± 0.00	1.45 ± 0.00	26.59 ± 0.13	15.58 ± 0.23	17.62 ± 0.27
3	24.26 ± 0.09	0.78 ± 0.00	1.46 ± 0.00	26.51 ± 0.19	15.48 ± 0.13	17.23 ± 0.31
4	26.09 ± 0.94	0.85 ± 0.01	1.61 ± 0.01	25.65 ± 0.30	14.58 ± 0.37	16.67 ± 0.48
5	28.55 ± 0.40	0.85 ± 0.00	1.60 ± 0.01	25.45 ± 0.13	13.84 ± 0.14	15.95 ± 0.31
Degradation rate				16.13%	14.24%	13.91%

Values represent mean ± SD (n = 3).

^1^Blank was initial switchgrass without adding strain.

^2^Determination of the glucose and xylose content in the fermentation broth following complete acid hydrolysis and calculated them into the content of cellulose and xylan.

Cultivation time with the bacteria progressively decreased switchgrass weight, while switchgrass particle size reduced and culture fluidity increased. Over 5 days, switchgrass cellulose and xylan hemicellulose degradation were 16.13 and 14.24%, respectively. Acid-insoluble lignin degradation was 13.91% over this period. In addition, acid environment also can make sugar hydrolyzed in the culture liquid. Cellulose and xylan degradation was also determined relative to the total dry weight. Finally, switchgrass hydrolyzation by *B. subtilis* 1AJ3 and its energy consumption remained relatively.

### Analysis of lignin degradation by gas chromatography–mass spectrometry

Since lignin content decreases during switchgrass degradation, we used the GC-MS to verify lignin degradation by *B. subtilis* 1AJ3. As lignin bears a benzene ring, analysis of benzene ring-bearing compounds is commonly used to detect the lignin content. GC-MS quantified low molecular weight benzene ring-containing compounds after pretreatment of switchgrass sample and methylsilylation. The total electron flow diagram (TIC) is shown in Supplementary File 1. Aromatic compounds corresponding to each component were identified in the NIST database. The peak areas of the different components were integrated, and the total area of all aromatic compounds was set to 100%. The proportion (%) of different benzene ring-containing compounds was then determined as respective peak areas relative to the total peak area (Table 2).

**TABLE 2:** Identification of aromatic compounds in control sample and the fifth day degraded switchgrass sample by *B. subtilis* 1AJ3 as TMS derivatives.

RT^1^	CAS	Compound name	Mol Wt^2^	Formula	Chemical formula	Control Area^3^%	1AJ3 Area%
9.983	90-05-1	2-Methoxyphenol	124	C_7_H_8_O_2_	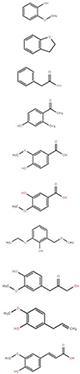	−	14.18
12.725	496-16-2	Coumaran	120	C_8_H_8_O		−	18.54
13.508	103-82-2	Benzeneacetic acid	136	C_8_H_8_O_2_		−	31.53
15.452	875-59-2	Ethanone,1-(4-hydroxy-2-methylphenyl)-	150	C_9_H_10_O_2_		−	24.43
21.469	121-34-6	Vanillic acid	168	C_8_H_8_O_4_		−	4.94
21.504	645-08-9	Isovanillic acid	168	C_8_H_8_O_4_		60.86	−
23.721	0-00-0	2-Ethoxy-6-(methoxymethyl)phenol	182	C_10_H_14_O_3_		−	3.08
25.383	4899-74-5	1-Hydroxy-3-(4-hydroxy-3-methoxyphenyl)acetone	196	C_10_H_12_O_4_		17.96	−
26.140	501-19-9	3-Allyl-6-methoxyphenol	164	C_10_H_12_O_2_		10.37	3.31
28.552	537-98-4	*Trans*-ferulic acid	194	C_10_H_10_O_4_		10.81	−
Sum						100	100

^1^RT, retention time.

^2^Mol Wt, molecular weight.

^3^“–” instead of did not detect.

The total amount of all detected aromatic components in each sample in the list is 100%. “Area%” instead of the component percent in each sample.

As aromatic compounds are the main lignin-degradation-related components (Zou et al., 2011), analysis of the aromatic compounds containing benzene rings in *B. subtilis* 1AJ3 vs. blank samples revealed that large aromatic compounds were broken into smaller molecular weight components (Ma et al., 2020b). Large molecular weight aromatic compounds, isovanillic acid (CAS: 645-08-9), 1-hydroxy-3-(4-hydroxy-3-methoxyphenyl) acetone (CAS: 4899-74-5), and trans-ferulic acid (CAS: 537-98-4) were completely broken into smaller components after cultured with *B. subtilis* 1AJ3 for 5 days. Component 3-allyl-6-methoxyphenol (CAS: 501-19-9) was partially degraded, and its content was significantly lower in the switchgrass sample after being cultured with the strain. In addition to the aromatic components’ reduction, new aromatic components are also produced, including 2-methoxyphenol (CAS: 90-05-1), coumarin (CAS: 496-16-2), phenylacetic acid (103-82-2), 1-(4-hydroxy-2-methylphenyl) ethanone (CAS: 875-59-2), vanillic acid (CAS: 121-34-6), and 2-ethoxy-6-(methoxymethyl) phenol (0-00-0).

### Analysis of switchgrass degradation by field emission scanning electron microscopy

FE-SEM was used to image switchgrass before and after culture with *B. subtilis* 1AJ3 (Figure 2). This analysis found that switchgrass progresses from smooth and compact, to completely fragmented, indicating that the bacteria had significantly degraded the switchgrass.

### Heat-resistance of *Bacillus subtilis* 1AJ3 and biomass degradation under acid-heat conditions

**FIGURE 3:**
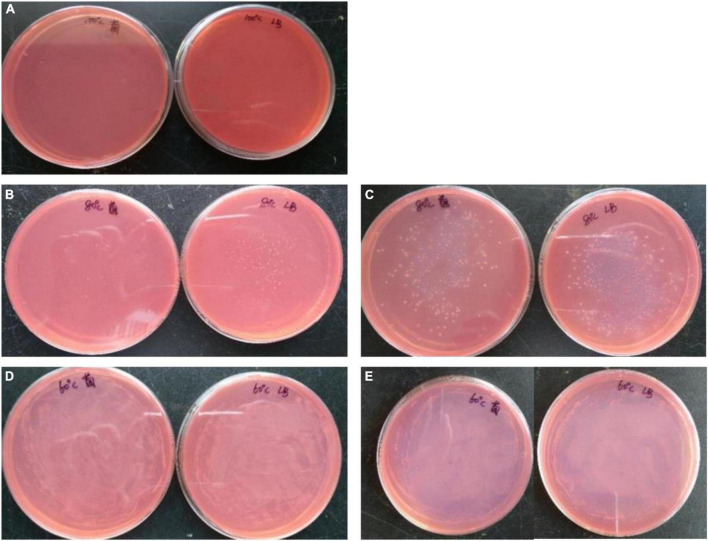
Growth situation of strain 1AJ3 on CMC-Na plate under different temperatures. **(A)** For 100°C; **(B,C)** for 80°C after 1st and 2nd day; **(D,E)** for 60°C after 1st and 2nd day.

Although *B. subtilis* 1AJ3 cannot grow under 100°C, it could tolerate up to 80°C, and some colonies grew within 24 h. Moreover, the most surprising finding was that *B. subtilis* 1AJ3 thrive at 60°C (Figure 3). We have previously evaluated influence of the media’s initial pH on *B. subtilis* 1AJ3 and found that the bacteria can grow at low pH (4.0) with high levels of reducing sugar accumulated (Ma et al., 2020a). Because the low pH environment also could hydrolyze some cellulose, an acid-heated growth condition for the strain to degrade biomass cellulose is preferred.

Two hydrolyzed conditions by strain 1AJ3 were applied to biomass raw materials. None of the raw materials was pretreated, but the treatment process was performed at a high temperature to reduce the adhesion of raw materials, suppress other microorganisms, and minimize their impact on the experiment. Since reducing sugar content in the culture reaches dynamic equilibrium in 3 days, in this experiment, the reducing sugar content was determined on the third day. Reducing sugar content under different conditions was compared. Then, the feasibility of various biomass lignocellulose for industrial ethanol fermentation using strain 1AJ3 under different degradation conditions was evaluated.

**FIGURE 4:**
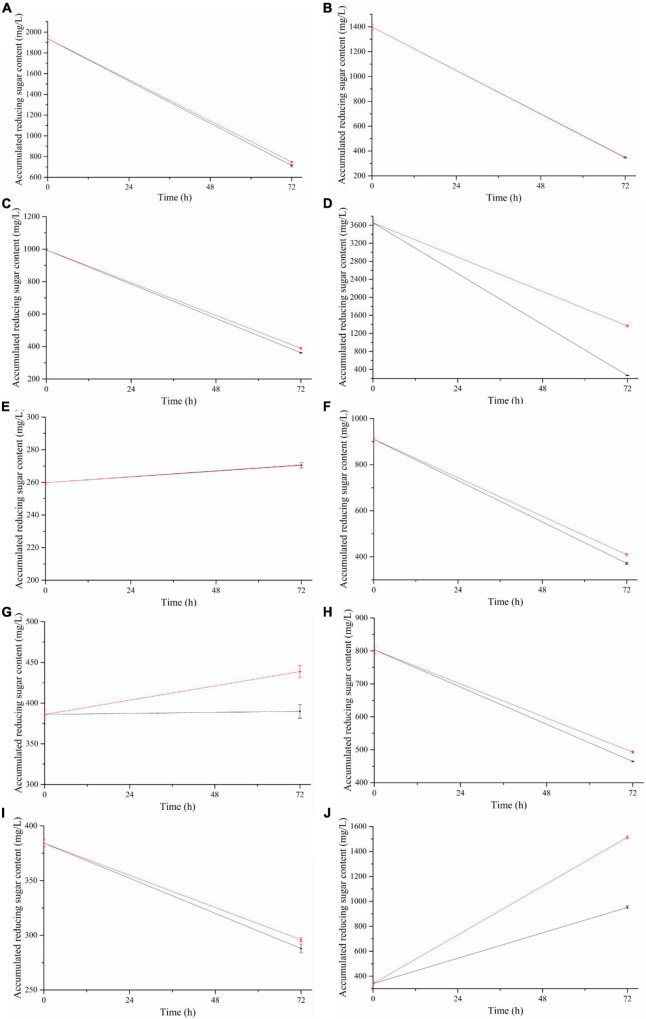
Accumulation of reducing sugar in 1AJ3 strain degraded at different unpretreated biomasses under two different conditions for 72 h. Black line for condition 1 of 37°C, pH 7.0, and red line for condition 2 of 50°C, pH 4.0. Biomass lignocellulose: **(A)** switchgrass, **(B)** wheat straw, **(C)** corn stalks, **(D)** corn cob, **(E)** rice husk, **(F)** sugarcane stalk, **(G)** pea straw, **(H)** ginger stems and leaves, **(I)** peanut shells, and **(J)** coffee ground.

Under the two conditions tested, *B. subtilis* 1AJ3 had almost no effect on wheat straw (Figure 4B) and rice husk (Figure 4E). However, the reducing sugar content produced from switchgrass (Figure 4A), corn straw (Figure 4C), sugarcane straw (Figure 4F), ginger in straw (Figure 4H), and peanut hull (Figure 4I) was slightly higher when using condition 2 than condition 1. Condition 2 produces significantly higher levels of reducing sugar from corn cob (Figure 4D), pea straw (Figure 4G), and coffee grounds (Figure 4J). Under the same culture rotation speed and time, when the bacteria’s degradation of cellulose to produce reducing sugar and its consumption of reducing sugars are at dynamic equilibrium, condition 2 (50°C, initial pH 4.0) is more conducive for biomass degradation relative to condition 1 (37°C, initial pH 7.0).

The character of raw material affects reducing sugar content, and corn cob is the richest, followed by switchgrass, and wheat straw, with rice husk being the poorest. However, no matter how high the reducing sugar content in the culture is during the initial state, cultivation with the bacteria eventually reaches a relatively stable reducing sugar level. Coffee grounds treated under condition 2 attained the highest sugar content (1513.58 mg/L), followed by the corncob in condition 2 (364.51 mg/L) (Figure 4D), coffee grounds in condition 1 (1951.91 mg/L) (Figure 4J), and switchgrass (Figure 4A) in condition 2 and condition 1 (747.83 mg/L and 714.40 mg/L, respectively). Reducing sugar content was below 300 mg/L for rice husk and peanut hull in both conditions, while corn cob produced < 300 mg/L in condition 1.

The reducing sugar content utilization by the bacteria for growth was greater than the reducing sugar produced by its degradation of cellulose, resulting in a downward trend (Figure 4). However, rice husk (Figure 4E), pea straw (Figure 4G), and coffee grounds (Figure 4J) showed an upward trend, and coffee grounds showed an upward trend in both conditions.

### Expression and purification of recombinant Bgl-16A

The *bgl*-16A gene sequence (732 bp) showed that it encoded 244 amino acids. The recombinant enzyme Bgl-16A containing a His-tag at the C-terminal, with a totally 252 amino acid sequences, was submitted to the NCBI database with GenBank access number QIP68091.1. The amino acid sequence blast showed that the enzyme has 99.08% identity with and 86% coverage of the sequence of beta-1,3-1,4-glucanases (PBD id: 3O5S), which belongs to the glycosyl hydrolase (GH) family 16 (EC number 3.2.1.73).

**FIGURE 5:**
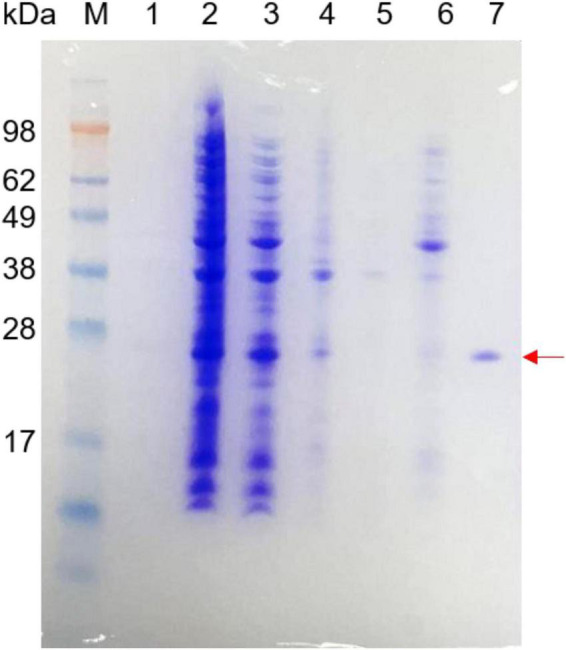
SDS-PAGE of purified recombinant Bgl-16A protein. Line 1: supernatant of fermentation liquid; Line 2: all cells after ultrasonically broken; Line 3: supernatant of cells after ultrasonic fragmentation; Line 4: precipitate of cells after ultrasonic fragmentation; Line 5: flow liquid through the Ni-NTA column; Line 6: 40 mM imidazole eluent; Line 7: purified enzyme obtained by 200 mM imidazole eluent liquid.

The recombinant beta-glycosidase hydrolase was expressed in *E. coli* and was purified using a Ni-NTA column. After removing the impure protein using 40 mM imidazole, 200 mM imidazole was used for purified protein. The purified recombinant Bgl-16A and the purification process are shown in Figure 5. The molecular weight (*Mw*) of purified Bgl-16A was about 26 kDa, consistent with the predicted molecular weight. The purified recombinant Bgl-16A showed a high enzymatic activity of 365.29 ± 10.43 U/mg.

### Characterization of recombinant Bgl-16A

**FIGURE 6:**
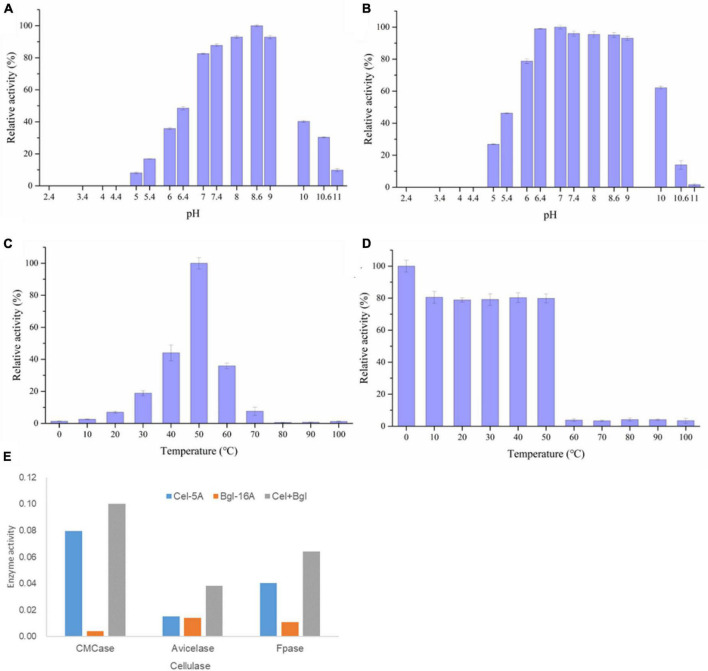
Characterization of Bgl-16A. **(A)** Optimum pH; **(B)** pH stability; **(C)** optimum of temperature; **(D)** temperature stability; **(E)** synergy effect of Bgl-16A and Cel-5A.

The enzymatic and physiological characterization of the recombinant Bgl-16A at different pHs and temperatures showed that the optimal pH was 8.6 (Figure 6A) and the temperature was 50°C (Figure 6C). More than 80% of maximum enzyme activity was reached between pH 7.0 and 9.0, indicating that Bgl-16A was suitable for neutral and slightly alkaline reaction conditions. At pH levels under 4.5, the recombinant protein precipitates and activity loss were observed. The optimal stability pH for Bgl-16A lies between 6.4 and 9.0 (Figure 6B), with relative enzyme activity over 93%. The optimal temperature of Bgl-16A was 50°C; therefore, it is an enzyme that needs strictly temperature. At temperature above or below 50°C, it decreased the enzyme activity under 50%. No enzyme activity was detected over 60°C for 30 min (Figure 6D). Bgl-16A did not exhibit any thermostable characteristics under high temperatures but contained a stable activity under low temperatures.

The effect of metal ions on recombinant Bgl-16A beta-glycoside hydrolase activity is reported in Table 3. Nearly, no metal ion increased enzyme activity. In comparison, Ca^2+^ could reduce enzyme activity at different concentrations. SDS also did not influence enzyme activity. EDTA could inhibit enzymatic activity at a high density (over 5 mM) while a low density (1 mM) did not. In addition, the synergy effect suggested that Bgl-16A and endo-cellulase Cel-5A had synergy action in CMCase, Avicelase, and FPase, with DS values of 1.195, 1.303, and 1.247, respectively (Figure 6E).

**TABLE 3:** Effect of metal ions on Bgl-16A.

Metal ion	Relative activity (%)		
	10 mM	5 mM	1 mM
Na^+^	107.14 ± 0.40	98.94 ± 2.58	106.98 ± 2.81
K^+^	87.38 ± 0.11	86.16 ± 0.11	84.66 ± 0.40
Cu^2+^	95.82 ± 1.26	93.71 ± 0.00	93.67 ± 0.17
Mg^2+^	103.82 ± 5.22	96.22 ± 4.13	94.64 ± 2.93
Zn^2+^	105.28 ± 0.52	105.40 ± 1.72	105.93 ± 0.52
Mn^2+^	92.61 ± 1.78	85.83 ± 1.03	87.70 ± 3.44
Fe^2+^	104.34 ± 1.38	96.59 ± 1.66	94.72 ± 0.29
Fe^3+^	102.40 ± 1.26	94.93 ± 0.92	94.56 ± 0.40
Ca^2+^	73.98 ± 0.11	81.24 ± 2.35	86.32 ± 1.15
[NH4]^+^	101.95 ± 4.99	102.52 ± 3.50	98.17 ± 0.57
SDS	100.20 ± 0.11	100.65 ± 0.40	98.82 ± 1.61
EDTA	46.98 ± 3.50	60.22 ± 3.50	97.28 ± 2.41

Enzyme activity without metal ions was set as 100%.

### Sequence analysis, homology model, and molecular docking

The recombinant enzyme comprises 252 amino acids, including the His-tag. The theoretical pI and molecular weight were determined as 6.79 and 28.55 kDa, respectively. Bgl-16A has a GH16 domain spanning from amino acid 35–242, presenting a beta-sandwich structure. The homology model is based on AlphaFold 2. The catalytic residues’ active sites were 134E and 138E according to the structure of 3O5S in PBD database. The protein displayed the typical structure of two juxtaposed curved anti-parallel beta-sheets as in the previous study (Furtado et al., 2011). However, the previous study only considered lichenan as the enzyme hydrolyzation substrate to examine beta-1,3-1,4-glucanase and used bis–tris-propane molecule for molecular docking.

**FIGURE 7:**
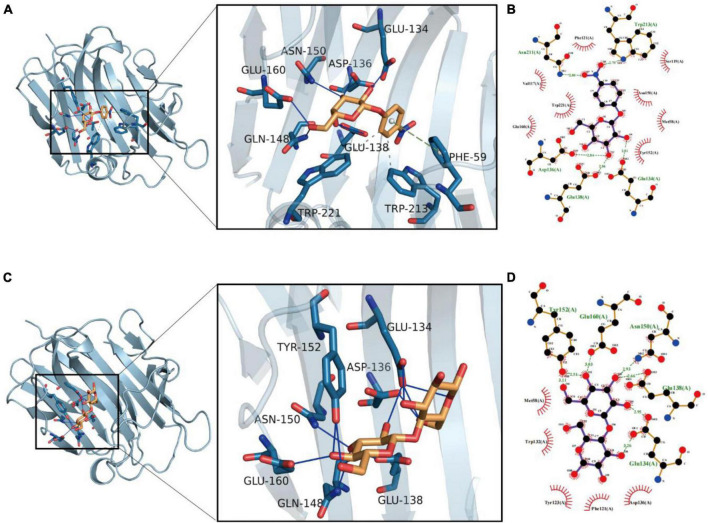
Molecular docking results of Bgl-16A with substrates: **(A)** molecular docking with p-NPG and **(B)** interaction with amino acids; **(C)** molecular docking with cellobiose and **(D)** its interaction with amino acids. Molecular docking results of Bgl-16A with substrates: **(A)** molecular docking with p-NPG and **(B)** interaction with amino acids; **(C)** molecular docking with cellobiose and **(D)** its interaction with amino acids.

The molecules were docked 100 times and divided into different groups using RMSD value until the end of docking. Cluster analysis of molecular docking results shown in Supplementary File 2 indicates that p-NPG has the better docking and clustering, when compared to cellobiose. The p-NPG can stably bind to the Bgl-16A cavity, and their binding energy is –5.72 kcal/mol. An AutoDock scoring value lower than –7.0 indicates that the target has a strong binding ability with the compound. In contrast, a score value between –5.0 and –7.0 indicates that the small molecule of the compound has a good binding ability with the target, and a score value between –5.0 and –4.25 indicates that the compound has a certain binding ability with the macromolecule target. The p-NPG mainly acted through hydrophobic interactions, hydrogen bonds, and π-stacking (precipitate) interactions. As shown in Figure 7A, p-NPG could form hydrophobic interactions with TRP221 and TRP213 of the protein. The hydroxyl group could form stable hydrogen bonds with ASP136, GLU138, GLN148, ASN150, TYR152, and GLU160 as hydrogen bond donors. The benzene ring also can form π-stacking (stacking) conjugated interaction with PHE59 (Figure 7B).

Cellobiose can stably bind to the protein cavity with a binding energy of –4.78 kcal/mol. The cellobiose molecule mainly interacts through hydrophobic interaction and hydrogen bond interaction. As shown in Figure 7C, the compound can form hydrophobic interactions with MET58, TRP132, TYR123, and PHE121. The hydroxyl group of the compound can act as hydrogen bond donor to form stable hydrogen bonds with GLN134, ASP136, GLU138, GLN148, ASN150, TYR152, and GLU160 of the protein, which is the main force promoting its binding to the active site (Figure 7D).

### Potential application of *Bgl*-16A in lignocellulose pretreatment

To further explore the application of Bgl-16A in lignocellulose or biomass waste, lignocellulosic switchgrass was used as the substrate to detect the hydrolyzation and saccharification abilities. In addition, coffee grounds were applied as the substrate to widen the application range. The crude enzyme was used for this part of the experiment.

**FIGURE 8:**
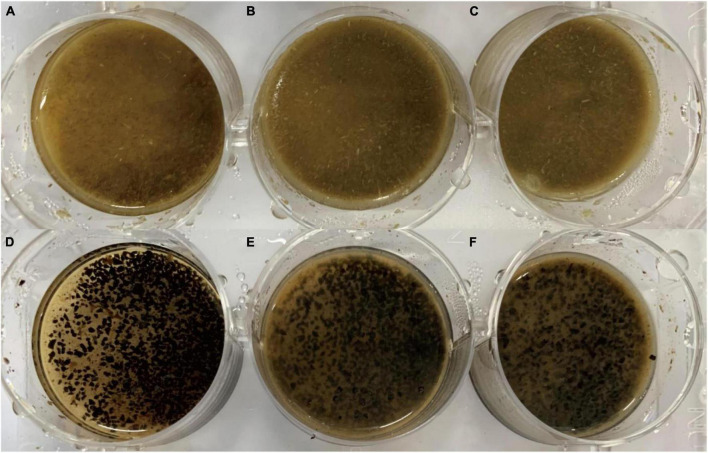
Application of recombinant Bgl-16A in 5% (w/V) switchgrass and coffee grounds. **(A–C)** Was for switchgrass with Bgl-16A content of 10, 20, and 40% (v/v) in 20 h; **(D–F)** for coffee grounds with Bgl-16A content of 10, 20, and 40% (v/v) in 20 h.

After enzyme culturing for 20 h, the hydrolyzation phenomenon was evident. The thick cellulose particles became fine and loose, and lignocellulose became thinner when the enzyme amount was increased (Figures 8A–C). This was particularly true for coffee grounds, whereby the dark brown crushed granules turned into light-colored loose tiny particles (Figures 8D–F). The total reducing sugar content of the switchgrass (coffee grounds) showed a saccharification rate of 10.40% (3.18%) of the total weight with 40% recombinant Bgl-16A. This demonstrates the high reducing sugar content of the switchgrass hydrolyzation. Switchgrass hydrolyzation had a higher reducing sugar content than coffee grounds. However, the hydrolyzation and degradation also predicted the challenge of the coffee grounds waste industry.

## Discussion

Biomass biodegradation by bacteria is widely researched and applied for its easy genome operation and strong environment tolerance (Carlos et al., 2018). Here, we evaluated the ability of *B. subtilis* 1AJ3 to degrade switchgrass lignocellulose and examined switchgrass cellulose, hemicellulose, and lignin degradation. To satisfy the strain growth temperature with the optimum degradation condition of lignocellulose, the strain 1AJ3 observed can grow under high temperatures. It showed that acid-heat conditions could provide a more suitable environment for a strain to hydrolyze cellulose. Afterward, a beta-glycoside hydrolase was cloned from the strain and overexpressed in *E. coli*. Furthermore, the enzyme was proved temperature-sensitive and only functioned at 50°C, which also explained the better degradation of biomass by the strain 1AJ3 under acid-heat conditions.

Wild-type bacteria have been screened for lignocellulose degradation as they are an important resource for the reducing sugar during biomass biodegradation (Ahmed et al., 2018; Puentes-Tellez and Salles, 2020; Liu et al., 2022). In this study, *B. subtilis* 1AJ3 can degrade switchgrass over 5 days during which switchgrass granularity became thinner and smaller, and the degradation rates of cellulose, hemicellulose, and acid-insoluble reached 16.13, 14.24, and 13.91%, respectively. Although *B. subtilis* 1AJ3 efficiency is low relative to fungi (Yang et al., 2009), it still showed better cellulose degradation ability than some bacteria, even co-cultured microbes. The bacteria *Rhizobium sp.* YS-1r hydrolyze switchgrass cellulose, xylan, and acid-insoluble lignin at 30°C with degradation rates of 19.33, 6.56, and 15.06%, respectively; however, the process took 4 months (Jackson et al., 2017). Co-cultivation of the fungi *Aspergillus niger* and *Trichoderma viride* to degrade untreated corn stover attained cellulose degradation rates of about 40% needs 35 days (Youzhou et al., 2015). So, *B. subtilis* 1AJ3 achieves a greater lignocellulose degradation rate in terms of shortened time.

Scanning electron microscopy was used as a direct observation method of switchgrass degradation by *B. subtilis* 1AJ3. SEM revealed that switchgrass integrity was fragmented, making the edges rough and irregular relative to their undegraded state. Wheat straw (Kshirsagar et al., 2016) and switchgrass (Wang et al., 2017) have been reported to degrade by other bacteria in similar manner. Lignin is regarded as the key factor in cellulose degradation. Because lignin wraps outside cellulose, it greatly stabilizes its structure and reduces contact between cellulose and cellulase or the microorganism (Sun et al., 2014), making cellulose hard to degrade (Brown and Chang, 2014). Bacterial strains that disrupt lignin structure during cellulose degradation can increase their contact area with cellulose. Here, we evaluated lignin degradation by GC-MS (Chen et al., 2012). High molecular weight aromatic compounds that contain a benzene ring are degraded to low molecular weight aromatic compounds. Thus, lignin degradation by *B. subtilis* 1AJ3 is simultaneous with cellulose degradation.

As the cellulases play roles through synergy, the optimum active conditions can improve the enzymes’ hydrolyzation effect. Here, based on the acid (Ma et al., 2020a) and heat resistance (evaluated in this study) of *B. subtilis* 1AJ3, two conditions were set to examine the bacteria’s performance against various biomass raw materials by quantifying the total reducing sugar content. These features expand its industrial application range. Raw material pretreatment has always been a major bottleneck in the degradation process of lignocellulose due to its high-temperature conditions (Chen and Qiu, 2010; Mishra and Ghosh, 2017). Studies on thermophilic microorganisms have shown that their biomass degradation ability requires their optimal temperature conditions (Strang et al., 2017; Bibra et al., 2018), which can reach 80°C (Zayulina et al., 2019) and strain 1AJ3 also can stand these high temperatures. It promised that an acidic environment might promote straw biomass degradation, highlighting the potential value of acid-heat resistant strains in industrial applications. The acid-heat condition was more helpful than the natural condition for biomass degradation. Besides the initial pH can affect degree of cellulose hydrolyzation, more influence brought from the temperature factor.

Cellulose degradation strains would have substrate specificity according to cellulase secretory and the mutual synergistic effect with different enzymes. Similar to previous study (Sadalage et al., 2020), bacteria 1AJ3 can degrade more kinds of biomass. In addition, different carbon sources also affect the hydrolyzation by cellulases, and different degradation patterns and different substrates are also observed (Dou et al., 2019), indicating the necessity and importance of finding different strain’s optimum substrates. This analysis found that *B. subtilis* 1AJ3 has superior performance under acid-heat conditions, especially against corncobs, pea stalks, and coffee grounds. In addition, the bacteria exhibited the superior capacity to degrade coffee grounds, where the reducing sugar content produced from cellulose degradation was higher than the bacteria’s energy consumption, and the concentration of reducing sugar was highest (1513.58 mg/L), which also predicted that coffee grounds could be used as substrate. Regarding the final concentration of reducing sugars in the culture, switchgrass, corncob, and coffee grounds have the potential for industrial application.

Cellulases complete the hydrolysis process from cellulose to reducing sugar through synergistic action. While endo- and exo-cellulase respond to cutting long cellulose chains into shortened chains even cellobiose, beta-glycoside hydrolase plays important role in last steps obtaining monosaccharide. Beta-glycoside hydrolase Bgl-16A performance narrows optimum temperature range as in previous research (Furtado et al., 2011) that only 50°C could display high hydrolyze efficiency. It also explains why strain 1AJ3 degraded biomass more efficiently at temperature culture conditions: the beta-glycoside hydrolase and other cellulases (Yang et al., 2016; Ma et al., 2020a) could perform better. Although the temperature limit is strict, the optimum pH range will make Bgl-16A broaden its potential application, combined with no significant ion inhibition for enzyme activity. The degradation of coffee grounds also evidenced the high saccharification properties of the strain 1AJ3.

## Conclusion

The cellulolytic strain, *B. subtilis* 1AJ3, has the degradation ability against switchgrass lignocellulose and multi lignocellulose substrates (especially coffee grounds). Acid-heat culture conditions (50°C, pH 4.0) could increase the reducing sugar content in the reaction when biomass lignocellulose is inoculated with strain 1AJ3 due to the strain’s tolerance to high growth temperature. A beta-glycoside hydrolase belonging to the GH16 family with heterogeneous expression in *E. coli* was cloned, showing a strict optimum temperature of 50°C and enzyme activity up to 365.29 ± 10.43 U/mg, explaining why temperature affects lignocellulose degradation. This strain has a wide range of applications; therefore, more enzymes should be explored, and degradation of more lignocellulosic substrates should be attempted in future.

## Data availability statement

The original contributions presented in this study are included in the article/Supplementary material, further inquiries can be directed to the corresponding author.

## Author contributions

LM and XL designed the experiments and drafted the manuscript. LM and XW did the experiments and analyzed the data. JZ took part in revising the manuscript. All authors have read and approved the final manuscript.

## Footnotes

1
https://www.rcsb.org/
