# Supplementary material for: Degradation of switchgrass by Bacillus subtilis 1AJ3 and expression of a beta-glycoside hydrolase
Source: Front Microbiol. 2022 Jul 29;13:922371. doi: 10.3389/fmicb.2022.922371 (PMC9374367; doi:10.3389/fmicb.2022.922371)
Supplement: Supplementary file 1 [file Data_Sheet_1.docx]

Supplementary file 1


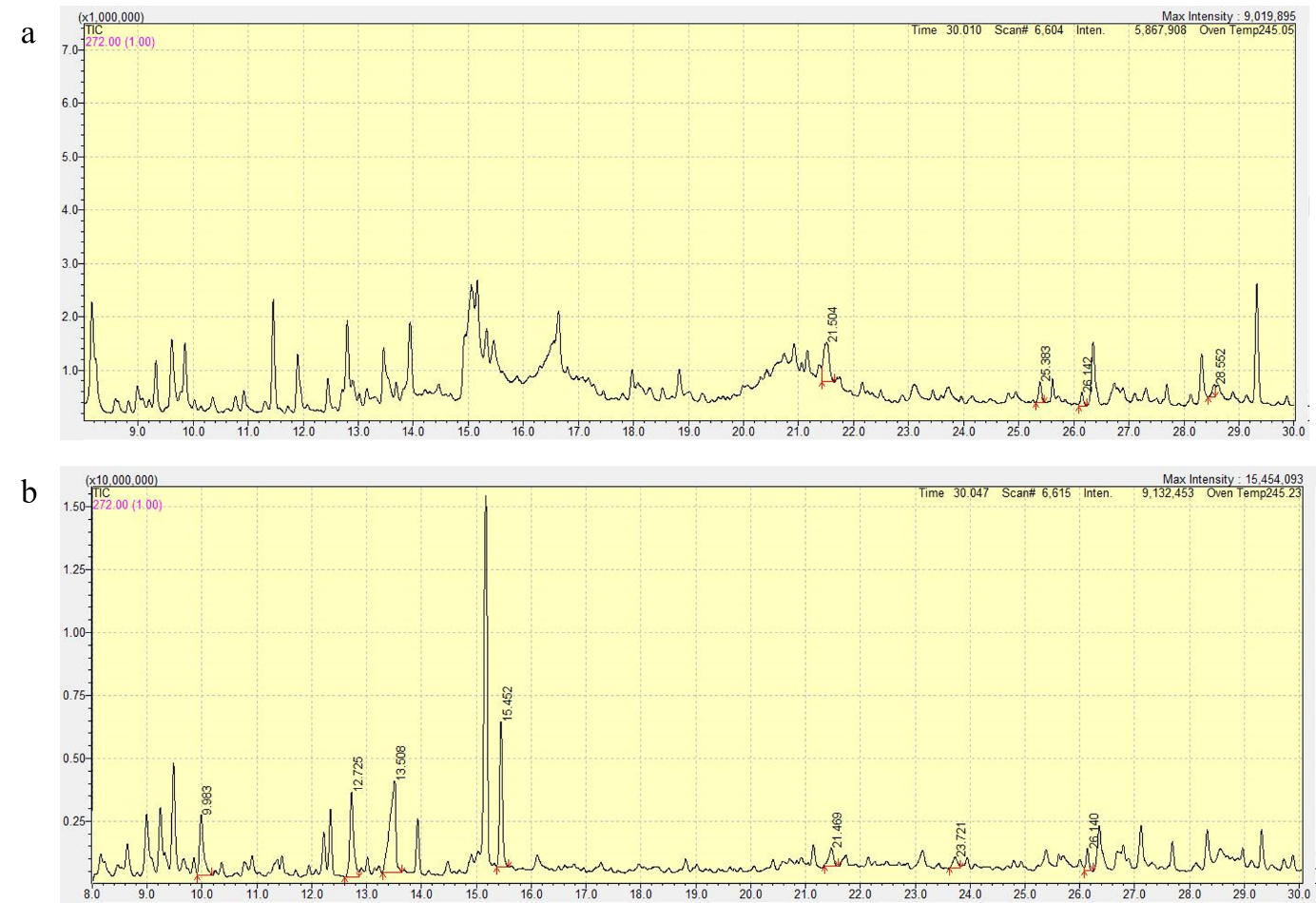


Fig S1: TIC of switchgrass degradation production by strain 1AJ3. a Blank b Sample after 5 days

Supplementary file 2


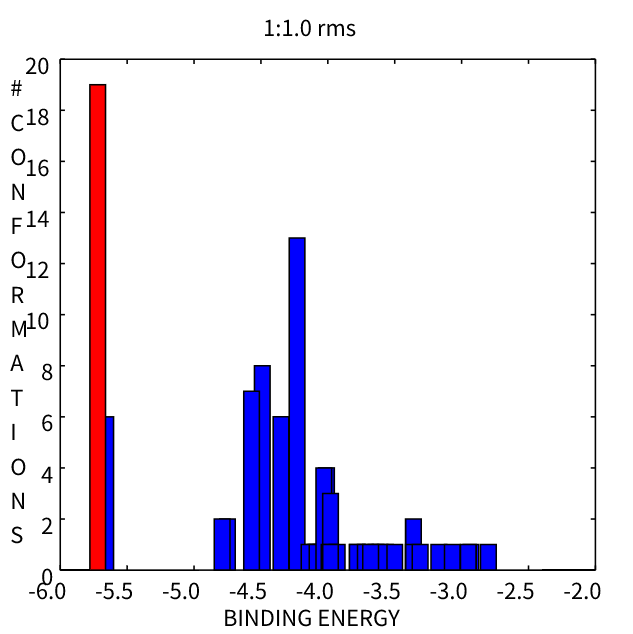

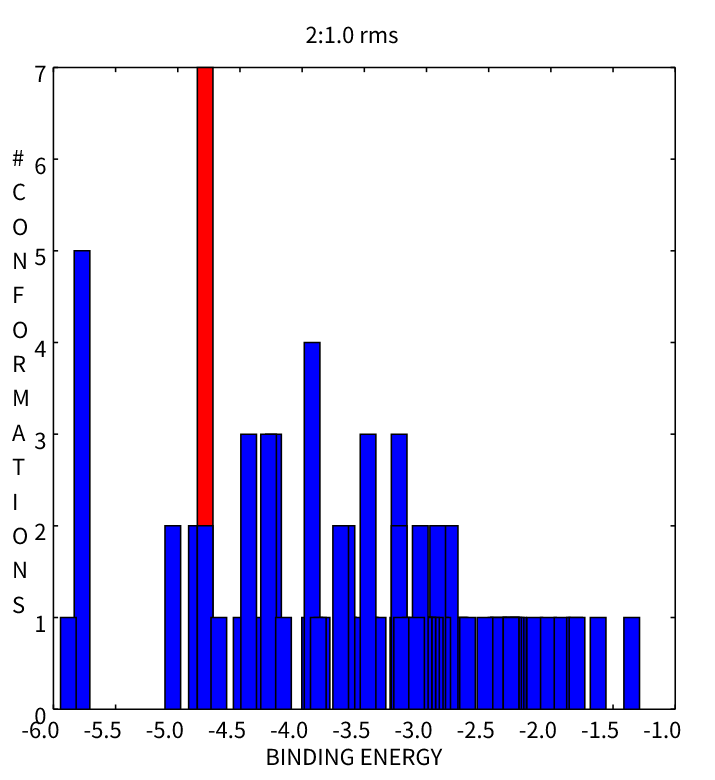


Fig. S2 Cluster analysis of molecular docking results of p-NPG and cellobiose
